# Clinical Implications of High Melioidosis Serology Indirect Haemagglutination Assay Titre: A 20-Year Retrospective Study from the Top End of the Northern Territory, Australia

**DOI:** 10.3390/pathogens14020165

**Published:** 2025-02-08

**Authors:** Cassandra Ho, Kevin Freeman, Celeste Woerle, Mila Mahoney, Mark Mayo, Robert W. Baird, Ella M. Meumann, Bart J. Currie

**Affiliations:** 1 Global and Tropical Health Division, Menzies School of Health Research, Charles Darwin University, Darwin, NT 0811, Australiaceleste.woerle@menzies.edu.au (C.W.); mark.mayo@menzies.edu.au (M.M.);; 2 Northern Territory Medical Program, Flinders University, Darwin, NT 0811, Australia; 3 Territory Pathology, Royal Darwin Hospital, Darwin, NT 0811, Australia; kevin.freeman@nt.gov.au (K.F.); rob.baird@nt.gov.au (R.W.B.); 4 Department of Infectious Diseases, Royal Darwin Hospital, Darwin, NT 0811, Australia

**Keywords:** melioidosis, *Burkholderia pseudomallei*, indirect haemagglutination assay, neglected tropical disease, serology, latency

## Abstract

Melioidosis, an infection with the bacterium *Burkholderia pseudomallei*, is highly endemic in the Top End of the Northern Territory of Australia. The indirect haemagglutination assay (IHA) is the most widely used serology test globally, but it is not standardised among the limited number of laboratories that perform it. While concerns have been raised about the sensitivity of IHA early in melioidosis infections, the advantage of IHA over more recently developed ELISAs is that testing serial dilutions allows a titre to be recorded. While in Australia a titre of 1:40 or higher is considered positive, the specificity at these low positive titres remains uncertain. However, a high titre is considered to represent recent or past true infection with *B. pseudomallei*, rather than cross-rection with other environmental *Burkholderia* species. Also, the natural history of IHA titres over time, in both asymptomatic infection and melioidosis has been little studied. We have assessed the clinical status and serology time courses of all 534 patients who had an IHA titre of 1:640 or higher, over a 20-year period. Of these, 324 (60.7%) were diagnosed with culture-confirmed melioidosis, with varying time courses of diagnosis of melioidosis in relation to the high serology. Of the 210 without confirmed melioidosis, 22 (10.5%) were considered highly likely to be melioidosis despite being culture-negative, and these were all treated as melioidosis. In the remainder, titres mostly gradually decreased over time, but the majority remained seropositive. A small number who had not been treated for melioidosis continued to have high IHA titres over years and activation from latency with a new diagnosis of melioidosis was occasionally documented. This study highlights the importance of a full clinical workup in those found to have high titre melioidosis serology as well as subsequent close clinical surveillance and where resources allow, yearly IHA in those not confirmed or treated as melioidosis.

## 1. Introduction

Melioidosis, a clinical disease resulting from infection with the Gram-negative environmental bacterium *Burkholderia pseudomallei*, is being increasingly recognised as endemic in tropical and subtropical regions globally [1]. Modelling estimated that there may be as many as 165,000 human cases of melioidosis per year worldwide, with an estimated 89,000 fatalities [2]. Despite this, a recent systematic analysis of the global burden of disease associated with 85 pathogens did not include melioidosis [3]. This analysis emphasised the observation of a substantial burden associated with “previously less recognised pathogens”, including the Gram-negative bacteria *Klebsiella pneumoniae*, *Escherichia coli*, *Pseudomonas aeruginosa* and *Acinetobacter baumannii* [3]. While a previously published systematic review of the global burden of melioidosis estimated the disability-adjusted life-years (DALYs) to be less for melioidosis than for the four Gram-negative bacteria noted above, the estimated melioidosis DALYs were substantially higher than the DALYs for many of the neglected tropical diseases (NTDs) formally listed by the World Health Organisation (WHO), including dengue, lymphatic filariasis, schistosomiasis and leprosy [4]. Calls for melioidosis to be added to the WHO list of NTDs seem justified [5].

Melioidosis has a wide range of clinical presentations and is notable as a great imitator of other diseases [6]. Pneumonia is the most common presentation, with a clinical spectrum from chronic illness mimicking tuberculosis, to acute community-acquired pneumonia, which can rapidly progress to fulminant septic shock with multiple abscesses in internal organs. Other presentations include skin infections, genitourinary infections, bacteremia with no evident focus, septic arthritis, osteomyelitis, muscle and soft tissue abscesses and neurological melioidosis [7]. Symptoms of those with melioidosis reflect both the foci of infection and the severity of illness, which is driven by patient clinical risk factors such as diabetes and by the mode of infection such as with inhalational melioidosis [1,6,7,8]. Most cases are acute presentations with an incubation period of 1–21 days (median 4 days) after the infecting event. Over half of patients are bacteremic, and septic shock requiring intensive care management has been documented for over 20% of all cases [7]. Nevertheless, in healthy children, the presentation may be without fever or systemic symptoms and with simply a single non-healing skin sore at the site of inoculation.

Diagnosis of melioidosis requires culture of *B. pseudomallei*, but a positive serological assay for antibodies to *B. pseudomallei* can heighten concern for melioidosis and direct further investigations and cultures to establish a diagnosis [1]. The indirect haemagglutination assay (IHA) can be particularly beneficial when screening patients who have migrated or travelled from melioidosis-endemic areas and present with a febrile illness that could be melioidosis, most commonly pneumonia [8].

The IHA remains the predominant serological test used in countries where melioidosis is endemic [9,10,11]. While the limitations of sensitivity (especially early in infection) and specificity (especially in endemic regions) of IHA are well recognised [9,10,11,12], it has an advantage over more recently developed enzyme-linked immunosorbent assays (ELISAs) in that an IHA titre is reported. A titre of 1:40 or higher is considered positive for infection with *B. pseudomallei* in Australia [10,11]. In endemic regions, most positive IHA results reflect past infection with *B. pseudomallei*, especially at low-positive titres [8]. Here we have analysed the clinical significance of an IHA titre of 1:640 or higher in a melioidosis-endemic setting.

## 2. Methods

The Darwin Prospective Melioidosis Study (DPMS) is run by the Royal Darwin Hospital (RDH) infectious diseases physicians and documents and manages all patients with culture-confirmed melioidosis in the tropical Top End of the Northern Territory of Australia, as previously described [7]. Patient demographic, epidemiological, clinical and laboratory details are stored prospectively in MariaDB v10.2.31 (Oracle, CA, USA).

The *B. pseudomallei* IHA is performed by Territory Pathology at RDH, as previously described [13]. Patient serum for testing is heat-inactivated at 56 °C and incubated with unsensitised ovine erythrocytes to remove non-specific agglutination. After centrifugation, the supernatant is then serially diluted (from 1:20 then doubling dilutions) and incubated with ovine erythrocytes sensitised to *B. pseudomallei* antigen produced from a combination of three local clinical isolates. The presence of antibodies is detected by observing red cell agglutination, with the highest titre at which red cell agglutination occurs recorded as the IHA titre result. Agglutination only in undiluted serum or only out to a dilution of 1:20 is a negative result in our laboratory. Red cell agglutination at a titre of 1:40 we report as positive, as are all higher dilution titres. While we report an IHA titre of ≥1:40 as positive, we note that in some laboratories in Thailand, only IHA titres of ≥1:160 are reported as positive. Unsensitised ovine erythrocytes are used as a control.

This study included prospectively collected DPMS data and retrospectively collected hospital demographic, clinical and laboratory data from all patients at RDH with an IHA titre of 1:640 or higher, between 1 October 2001 and 30 September 2021 (inclusive). For these high-titre patients, serial IHA titre results were followed where performed up to 30 September 2023, ensuring at least two years of follow-up after the initial high IHA for all those included in this 20-year study of high-titre IHA results. For the non-DPMS patients, their serial IHA titres were classified as either a single titre or two or more titres out to one or more of the following time periods: within 365 days (1 year), beyond 1 year out to 730 days (2 years), beyond 2 years out to 1825 days (5 years) and beyond five years. Serial serology results were categorised into the following three trends based on the most recent IHA titre: titres that remained high (1:640 and higher); titres that decreased but were still positive and titres that sero-reverted. A positive titre was defined as being 1:40 or higher. Seroreversion was defined as the last known IHA titre being <1:40.

For those patients with culture-confirmed melioidosis, the presentation was assigned as previously documented as one of acute melioidosis, chronic melioidosis (defined as symptoms for ≥2 months at the time of diagnosis) or activation from latency [7,14]. Activation from latency was as previously described and documented, using consensus criteria including clinical features, seasonality and past radiological imaging and serology results [7,14]. For those patients not confirmed as melioidosis, the clinical reason for the melioidosis serology testing was determined from the patient’s medical records as well as potential clinical risk factors for melioidosis such as documented diabetes and/or elevated HbA1c.

In addition to managing all patients with confirmed melioidosis, the RDH infectious diseases physicians assess all those with high melioidosis IHA titres, advising on further investigations and through consensus deciding those who require full therapy for melioidosis despite being culture-negative for *B. pseudomallei*. These “culture-negative melioidosis” patients receive intensive intravenous phase therapy and subsequent eradication therapy as per RDH guidelines and are followed up by infectious diseases physicians [7].

This study was approved by the Human Research Ethics Committee of Top End Health Service and Menzies School of Health Research (approval number 02/38 HREC).

## 3. Results

**Figure 1:**
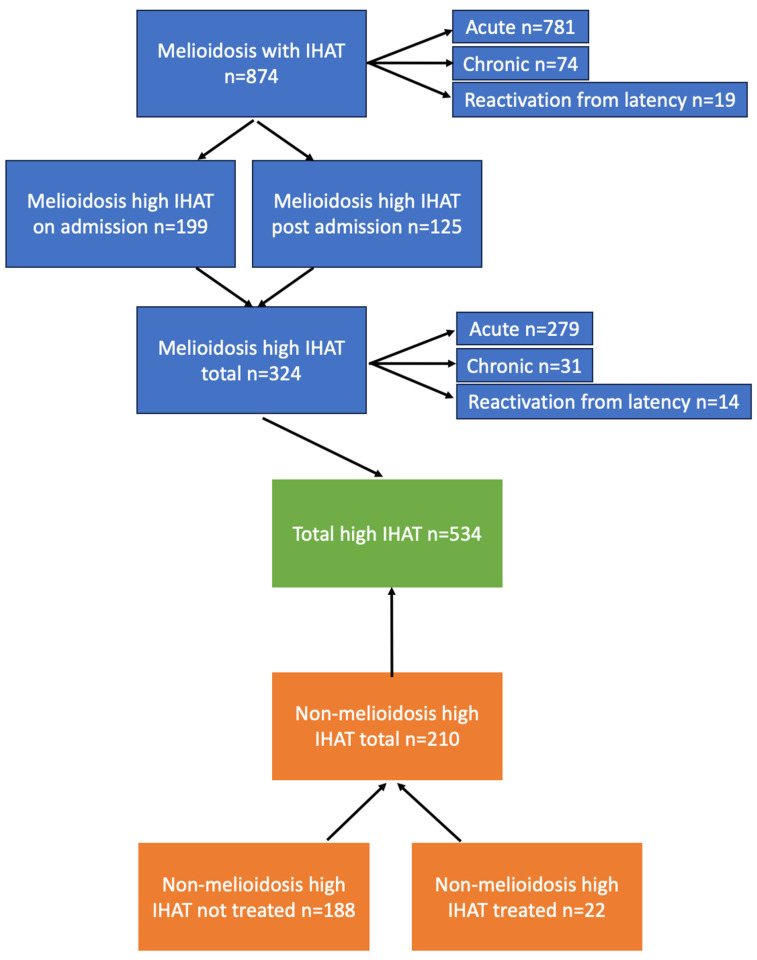
Schematic of 534 patients with melioidosis serology titre of 1:640 or higher.

Titres of 1:640 or greater accounted for 298 of 5323 (5.6%) consecutive IHA tests performed over a 12-month period in 2023. Over the 20 years between 1 October 2001 and 30 September 2021 (inclusive), there were 534 individuals with IHA titres of 1:640 and above (Figure 1). Of these, 324 (60.7%) were culture-confirmed melioidosis patients and 210 (39.3%) were culture-negative. Of the 210 who did not culture *B. pseudomallei*, 22 (10.5%) were assigned as “culture-negative melioidosis” by the RDH Infectious Diseases team and fully treated for melioidosis.

### 3.1. Melioidosis Cases

Over the 20-year period of the total 874 DPMS patients with culture-confirmed melioidosis and IHA testing, 324 (37.1%) had a titre of 1:640 and above (Figure 1). Of these, 199 (22.7% of DPMS cases) had titres of 1:640 and above on admission, while 125 patients (14.3%) had lower or negative titres on admission that subsequently rose to 1:640 and above after admission (Figure 1). As previously documented for the DPMS, sputum-culture-positive pneumonia was the commonest presentation, and bacteremia was present in 56% overall [7].

The 324 high-titre melioidosis patients comprised 279 (35.7%) of the 781 presenting with acute melioidosis, 31 (41.9%) of the 74 presenting with chronic melioidosis (symptoms for ≥2 months at the time of diagnosis) and 14 (73.7%) of the 19 patients considered to be activation from latency. As expected, IHA titres of 1:640 and above already by admission (rather than rising subsequent to admission) were seen in 12/14 (85.7%) of those with activation from latency and 26/31 (83.9%) of those presenting with chronic melioidosis. In contrast, of the 279 presenting with acute melioidosis, only 161 (57.7%) had IHA titres of 1:640 and above at admission and in the other 118 (42.3%) IHA titres rose to 1:640 and above subsequent to admission.

### 3.2. High Titre but Not Melioidosis

Of the 210 patients with IHA titres of 1:640 and above who were never culture-positive for *B. pseudomallei*, 188 (89.5%) did not receive definitive therapy for melioidosis. Table 1 shows their demographics, proportion with diabetes and the reasons for the melioidosis serology testing, noting that there were missing data for some parameters in this retrospective analysis. There was a wide age range (median 42; IQR 28 years) and slight male predominance (57.4%). Of those with data retrieved, 24.5% had diabetes and respiratory infections were the commonest reason for the serology testing being ordered.

Serial IHA titres were available for 132 of 188 (70.2%) and are shown in Table 2a. Overall, there was a steady decline in titres over time, most evident after 1 year, with the majority decreasing to under 1:640. However, seroreversion to titres <1:40 was uncommon and a small number remained with titres ≥1:640 beyond 5 years.

### 3.3. High-Titre, Culture-Negative for B. pseudomallei but Fully Treated as Melioidosis—“Culture-Negative Melioidosis”

There were 22 of the 210 patients with IHA titres of 1:640 and above who were never culture-positive for *B. pseudomallei* but who were assigned as “culture-negative melioidosis” by the RDH Infectious Diseases team and fully treated for melioidosis. There was also a wide age range (median 29; IQR 41) and male predominance (59.1%). Of those with data retrieved, 31.3% had diabetes and there was a diversity of infectious presentations that led to the serology testing being ordered (Table 1). There were no statistically significant differences in the clinical features between those high-titre *B. pseudomallei* culture-negative patients who were or were not treated as melioidosis.

Serial IHA titres were available for all but 1 of the 22 culture-negative patients who were treated for melioidosis, and these are shown in Table 2b. There was a steady decline in titres, with downward trends more marked than seen in those not treated for melioidosis and with all eventually decreasing to <1:640. While the majority with titres available beyond 5 years still remained seropositive, a greater proportion sero-reverted to titres <1:40 than in those not treated for melioidosis.

## 4. Discussion

The IHA indicates infection with *B. pseudomallei*, with most individuals being asymptomatic and only a minority developing the clinical disease, which is known as melioidosis. It is not known how many of those with asymptomatic seropositivity have viable but unculturable *B. pseudomallei*, with the potential for subsequent activation from latency to develop melioidosis [8]. However, such activation from latency is uncommon, representing under 3% of all cases in the DPMS, with the vast majority of melioidosis reflecting recent infection, which is in stark contrast to latency with activation seen in tuberculosis [14].

In patients with melioidosis, approximately half have a negative IHA on initial presentation, but of these, the majority do subsequently seroconvert within 1–4 weeks after admission [13]. Response to treatment is often monitored with declining IHATs, but there remains a proportion of patients with persistently positive titres following therapy and only a small number of these subsequently have confirmed relapsed melioidosis [7,13]. Background seroprevalence in the Darwin region has been estimated at around 3% [11], which is in stark contrast to seropositivity rates in Thailand as high as 50% [9,15].

An IHA titre of 1:640 or higher represents a high titre. An IHA of ≥1:160 is used in Thailand for positive serology of melioidosis [16], while in the NT and elsewhere in Australia, we consider an IHA titre of ≥1:40 as positive [10,11,13]. While the specificity of IHA for infection with *B. pseudomallei* is likely to be lower at low positive IHA titres such as 1:40, titres of ≥1:640 are considered very likely to represent definite *B. pseudomallei* infection at some point in the past [8]. A study in Thailand refuted previous suggestions that positive melioidosis serology often represents exposure to other environmental *Burkholderia* such as *B. thailandensis* [16]. Nevertheless, the natural history of individuals with these high titres has not been assessed in melioidosis-endemic regions.

In our setting, 324 of 534 (60.7%) of those with an IHA titre ≥1:640 over a 20-year period were culture-confirmed as melioidosis, noting that multiple investigations with repeat cultures are sometimes required to establish the diagnosis. An additional 22 patients who were culture-negative on the full investigation were fully treated as melioidosis by consensus decision of the RDH infectious diseases physicians, based on consideration of environmental exposure risk factors, clinical risk factors such as diabetes, clinical findings, exclusion of other pathogens and the IHA titre ≥:640. Bayesian latent class modelling in Thailand estimated that the sensitivity of culture for diagnosing melioidosis was only 60.2% [12], justifying clinical decisions to treat patients with melioidosis in selected culture-negative scenarios. Nevertheless, we undertake multiple rounds of culturing and from multiple clinical samples including rectal and throat swabs in those with high IHA titres before a final decision to continue and complete full therapy for melioidosis.

Overall, 324 of 874 (37%) patients with culture-confirmed melioidosis over the 20-year period had titres of ≥1:640 at some stage. Only 199 (22.8%) had titres ≥1/640 on admission, especially those with either chronic melioidosis or with considered activation from latency. The other 125 with initial negative or low titres rose to ≥1:640 subsequent to admission, mostly within 1–3 weeks.

The reason for testing in those with high titres but who were never confirmed as melioidosis was most commonly respiratory infections such as community-acquired pneumonia but also included those presenting with a range of other infectious disease syndromes potentially consistent with melioidosis, including skin and soft tissue infections and genitourinary infections.

The natural history of the IHA titres in those with titres ≥1:640 who did not have or did not develop confirmed melioidosis showed variable trends over time. For the minority who the infectious diseases team decided to fully treat as melioidosis, despite no confirmed culture of *B. pseudomallei*, a decrease in titres was most common, with some eventually sero-reverting to a negative IHA and none having a persisting titre of ≥1:640. However, some of those treated as melioidosis did have persistent positive IHA titres, which are also seen following treatment of confirmed melioidosis [13]. For those who were not treated for melioidosis, the proportion with high titres decreased more gradually and the majority remained seropositive. Seroreversion was seen in some, mostly when follow-up testing extended over a 5-year or longer period.

Our data provide support for our policy of a full clinical workup for melioidosis in those with high melioidosis serology. This includes cultures of blood, sputum and urine, with the use of selective agar and/or broth for non-sterile sites and also for throat and rectal swabs [1,7,8]. Imaging includes chest X-ray or chest CT scan and abdominal/pelvis CT scan or ultrasound. In those with high titre serology, subsequent yearly IHA is recommended, and where IHA titre remains persistently high, close clinical surveillance is important with a low threshold for repeat imaging and cultures. We restrict completion of full melioidosis treatment to a minority of those who have persistently high IHA but are culture-negative despite repeated clinical sampling. Such patients include those with infection in sites difficult to access for culture, such as neurological melioidosis, mycotic vascular aneurysms, mediastinal lymphadenitis and persistent internal organ abscesses.

There are important limitations to this study. While the patients with confirmed melioidosis are part of the Darwin Prospective Melioidosis Study, all other data were collected retrospectively. This limits the accuracy of the assessment of the clinical reasons for the serology testing. Importantly, the follow-up of patients over the 20 years was variable, resulting in attrition bias related to patients being lost to follow-up, as evident from the large number of patients with only a single high IHA titre. For the subset of patients who sero-reverted with titres monitored over 5 years, it is not possible to accurately assess the timing of seroreversion, given the often-limited number of IHA tests to analyse. Additionally, for the subset of non-melioidosis patients prescribed full treatment for melioidosis, it was difficult to assess if the full eradication therapy was completed, which may in part explain some of the persistently high or positive serology in the years following treatment. Finally, the relevance of our data and findings to melioidosis-endemic regions outside of Australia may be limited for two reasons: Firstly, each region or country prepares its own IHA using local isolates of *B. pseudomallei*, making comparisons of cut-offs for positivity and results between studies problematic. Secondly, resource limitations in many melioidosis-endemic regions limit the capacity for laboratory culture diagnosis of melioidosis, let alone serology and clinical follow-up of high-titre serology cases.

## 5. Conclusions

In summary, an IHA titre of 1:640 and higher reflected confirmed melioidosis in 60.7% of patients. Of these, the majority had acute melioidosis, where initial IHA was often negative but with subsequent seroconversion to a high titre. Around 10% (n = 31) of those with melioidosis and a high titre were patients with chronic melioidosis and only 14 (4%) high-titre melioidosis patients were thought to be activation from latency subsequent to prior infection with *B. pseudomallei*. While subsequent activation from latency is a rare event in those with positive melioidosis serology, it remains unknown what proportion of those with persisting high-titre serology still harbour viable *B. pseudomallei*. While those with high-titre positive serology often show decreased titres and sometimes seroreversion on subsequent serology, some have persisting high titres for more than five years and these patients need careful clinical and laboratory monitoring for activation from latency. Prospective studies are required to better evaluate the natural history of positive melioidosis serology in melioidosis-endemic locations.

## Figures and Tables

**Table 1:** High-titre culture-negative patients—not treated and treated for melioidosis.

	Not Treated for Melioidosis Number (%)	Treated for Melioidosis Number (%)
All patients	188 (89.5%)	22 (10.5%)
Age range (years)	4–102 (median 42; IQR 28)	5–72 (median 29; IQR 41)
Sex:		
Female	80 (42.6%)	9 (40.9%)
Male	108 (57.4%)	13 (59.1%)
Diabetes:		
Data missing	37	6
Present	37 (24.5%)	5 (31.3%)
Absent	114 (75.5%)	11 (68.8%)
Infectious focus that led to serology testing		
Data missing	48	4
Respiratory	41 (29.3%)	3 (16.7%)
Genito/Urinary	3 (2.1%)	1 (5.6%)
Skin/Soft tissue	26 (18.6%)	4 (22.2%)
Central nervous system	8 (5.7%)	1 (5.6%)
Osteomyelitis	1 (0.7%)	0
Septic arthritis	2 (1.4%)	1 (5.6%)
Other focus	24 (17.1%)	3 (16.7%)
No evident focus of infection	31 (22.1%)	5 (27.8%)
Immunosuppression screening	4 (2.9%)	0

**Table 2:** (**a**) Trends in serology over time for high-titre culture-negative patients not treated for melioidosis n = 188; (**b**) trends in serology over time for high-titre culture-negative patients treated for melioidosis n = 22.

(**a**)				
**Time Period**	**Single Titre**	**Remained High (≥640)**	**Decreased but Still Positive (≥1:40)**	**Sero-Reverted**
Single titre n = 56	56 (29.8%)			
All until 365 days n = 123		87 (70.7%)	34 (27.6%)	2 (1.6%)
All until 730 days (2 yrs) n = 58		26 (44.8%)	30 (51.7%)	2 (3.4%)
All until 1825 days (5 yrs) n = 49		15 (30.6%)	34 (69.4%)	0
All more than 1825 days (>5 yrs) n = 32		4 (12.5%)	26 (81.3%)	2 (6.3%)
(**b**)				
**Time Period**	**Single Titre**	**Remained High (≥1:640)**	**Decreased but Still Positive (≥1:40)**	**Sero-Reverted**
Single titre n = 1	1 (4.5%)			
All until 365 days n = 21		18 (85.7%)	3 (14.3%)	0
All until 730 days (2 yrs) n = 8		2 (25.0%)	5 (62.5%)	1 (12.5%)
All until 1825 days (5 yrs) n = 7		1 (14.3%)	5 (71.4%)	1 (14.3%)
All more than 1825 days (>5 yrs) n = 5		0	3 (60.0%)	2 (40.0%)

## Data Availability

Delinked data can be requested from the authors.
